# Primary costovertebral hydatidosis revealed by compressive myelitis: a case report

**DOI:** 10.11604/pamj.2026.53.86.51202

**Published:** 2026-02-16

**Authors:** Fouzia Boudiab, Dahab Ouhabi, Hajar Naciri Darai, Ali Benomar, Houyam Tibar, Wafa Regragui

**Affiliations:** 1 Department of Neurology B and Neurogenetics, Specialties Hospital, Ibn Sina University Hospital, Mohammed V University, Rabat, Morocco

**Keywords:** Hydatidosis, spine, costovertebral, compressive myelitis, case report

## Abstract

Osseous hydatidosis is a rare manifestation of echinococcosis, accounting for a small proportion of hydatid disease cases, with spinal involvement representing the most severe form due to the risk of neurological complications. We report the case of a 32-year-old woman living in a rural area who presented with progressive paraplegia secondary to spinal cord compression, leading to the diagnosis of isolated primary costovertebral hydatidosis. Spinal magnetic resonance imaging revealed an extensive multivesicular lesion involving the thoracic spine with extradural spinal cord compression and displacement of the adjacent hemithorax without pulmonary parenchymal involvement. Emergency surgical decompression with cyst excision was performed, and histopathological examination confirmed the diagnosis. Postoperative antiparasitic treatment with albendazole was initiated, resulting in a favorable neurological outcome. This case highlights the diagnostic challenges, imaging features, and therapeutic management of this exceptional localization and underlines the importance of early diagnosis to prevent irreversible neurological sequelae.

## Introduction

Osseous hydatidosis is a parasitic infection caused by the larval stage of *Echinococcus granulosus* and represents a rare manifestation of hydatid disease [1]. Vertebral involvement is uncommon but constitutes the most severe form due to the infiltrative nature of the disease and the frequent intraspinal extension leading to spinal cord compression [2]. This form is often associated with contiguous costal involvement [3]. Primary costovertebral hydatidosis is exceptional and poses significant diagnostic and therapeutic challenges. We report a case of primary costovertebral hydatidosis revealed by compressive myelitis, highlighting the epidemiological context, diagnostic difficulties, imaging findings, and management strategies of this rare entity.

## Patient and observation

**Patient information:** a 32-year-old woman from a rural area, living in precarious socio-economic and hygienic conditions, was admitted to the neurological emergency department. She raised livestock, particularly sheep, and had close and prolonged contact with dogs. Her medical history was unremarkable. She presented with a gradually progressive clinical course over four months, marked initially by lumbosciatica, followed by lower limb weakness and sphincter disturbances in the form of urinary retention, evolving in an afebrile context with preserved general condition.

**Clinical findings:** neurological examination revealed spastic paraplegia, more pronounced on the left side, brisk and diffuse deep tendon reflexes in the lower limbs, bilateral Babinski sign, and a sensory level at the D10 dermatome.

**Timeline of current episode:** the patient initially experienced nonspecific lumbosciatica that progressively worsened over several months. Neurological deficits appeared later, prompting hospital admission. Spinal imaging was performed upon admission, revealing a compressive lesion. Urgent surgical management was subsequently undertaken. Postoperative treatment was initiated, followed by gradual neurological recovery.

**Figure 1:**
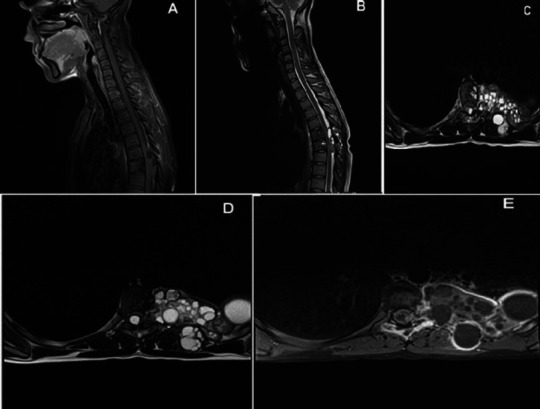
spinal magnetic resonance imaging: A) sagittal T1-weighted image, the lesions appear hypointense on T1-weighted images; B) sagittal T2-weighted image, hyperintense on T2-weighted images; C, D) axial T2-weighted images, hyperintense on T2-weighted images; E) post-contrast T1-weighted axial image: multilobulated lesion with both solid and multivesicular components, centered on the left costo-vertebral groove at the D6-D7 level, with peripheral contrast enhancement

**Diagnostic assessment:** spinal magnetic resonance imaging showed a multilobulated lesion with solid and multivesicular components, centered on the left costovertebral groove at the D6-D7 level. The lesions were hypointense on T1-weighted images and hyperintense on T2-weighted images, with peripheral contrast enhancement. The involvement extended to the D6 and D7 vertebral bodies, posterior vertebral arches, adjacent paravertebral muscles, and resulted in extradural spinal cord compression. A displacement of the left hemithorax was observed, without any associated pulmonary parenchymal lesion (Figure 1). The clinical presentation in a young woman living in a rural area, together with the imaging findings, strongly suggested the diagnosis of costovertebral hydatidosis. The extension workup, including chest radiography and abdominal ultrasound, revealed no additional hydatid localizations, allowing the diagnosis of isolated primary costovertebral hydatidosis. Hydatid serology was not performed. Laboratory investigations showed normal inflammatory markers and no eosinophilia.

**Therapeutic interventions:** the patient underwent urgent surgical decompression with excision of the compressive lesion. Histopathological examination of the surgical specimen confirmed the hydatid nature of the lesion. Postoperatively, antiparasitic medical treatment with albendazole was initiated for a duration of six months.

**Follow-up and outcome of interventions:** the postoperative course was favorable, with progressive and complete recovery of the sensorimotor deficit and no evidence of early recurrence.

**Patient perspective:** the patient reported a clear improvement in neurological symptoms after surgery and medical treatment. She expressed satisfaction with the care received and noted a significant improvement in mobility and daily activities.

**Informed consent:** it was obtained from the patient for publication of this case report and accompanying images.

## Discussion

Hydatidosis is a zoonotic parasitic disease caused by the development of the larval stage of *Echinococcus granulosus* in humans [1]. The liver (60-70%) and lungs (20-30%) are the most common sites of involvement, while atypical localizations, including the spleen, kidneys, heart, muscles, or bones, are much rarer [1]. Osseous hydatidosis is an uncommon form, accounting for only 0.5 to 2.5% of cases, with a predilection for the spine (44%), followed by the pelvis (16%), femur (15%), humerus (7%), and tibia (6%) [2]. The costo-vertebral form is exceptional, with an estimated frequency ranging from 0.18 to 1.21% [3]. Our patient presented with progressive spinal cord compression, without fever or general health deterioration. In the absence of trauma, such a clinical picture initially suggests tuberculous spondylodiscitis, particularly given the endemic context of tuberculosis in Morocco, although the patient showed no signs of systemic involvement. The rural environment, prolonged contact with dogs, close proximity to livestock, and poor hygiene conditions also supported a parasitic etiology, particularly vertebral hydatid cyst. A tumoral origin, whether benign or malignant, primary or metastatic, was also considered, although less likely given the absence of localized bone pain, tumor syndrome, and the preserved general condition. The epidemiological background suggestive of vertebral hydatidosis in our patient is well established in the literature. The parasite's life cycle involves two hosts: a definitive host, typically the dog, and an intermediate host, usually a sheep [1]. Humans are accidental hosts who get infected through ingestion of contaminated food or contact with infected dogs. Once ingested, the released larva penetrates the intestinal wall, enters the bloodstream, and migrates to various organs. The liver acts as the first capillary filter, followed by the lungs as the second, explaining the predominance of hepatic and pulmonary localizations. The rarity of osseous involvement may be attributed to the larger caliber of bone capillaries (18-22 µm) compared to those in soft tissues (10 µm), while the larva itself measures approximately 20 µm [4]. Bone involvement most often occurs hematogenously, after bypassing the hepatic and pulmonary filters, possibly via paradoxical embolism or through Batson´s vertebral venous plexus. Secondary involvement is also possible by direct extension from adjacent soft tissue foci.

In our patient, initial symptoms-nonspecific lumbosciatica evolving over several months, did not prompt early medical consultation. Neurological signs only appeared at a later stage. Such delayed diagnosis is frequently reported in vertebral hydatidosis [5]. In these cases, the initial pain is often intermittent, nonspecific, and may be mistaken for mechanical or degenerative lower back pain or sciatica. This latency is explained by the slow, infiltrative growth of the parasite, which progresses within bone tissue without forming a pericystic capsule or triggering marked inflammatory responses [2]. In the absence of compression on nearby structures and due to the low innervation of deep bone tissue, symptoms remain absent or misleading for extended periods, contributing to diagnostic delays in most cases. Clinically, as in our patient, the initial phase is marked by intermittent mechanical back pain, preserved general condition, absence of fever, and insidious evolution. Spinal cord compression is a serious and often revealing complication, which may occur gradually or acutely [2]. Topographically, the thoracic spine is most frequently involved (80%), followed by lumbar and sacral levels (18%). Cervical involvement is rare. The costo-vertebral form, as seen in our patient, is particularly rare and generally occurs through direct extension from an adjacent vertebral lesion [3]. Magnetic resonance imaging (MRI) is the imaging modality of choice for detailed assessment of vertebral hydatidosis [6]. Two main morphological patterns may be identified: the typical multivesicular form, with cysts appearing hypointense on T1-weighted images and hyperintense on T2-weighted images, without enhancement after gadolinium injection; and the pseudo-tumoral multilocular form, where internal septa appear hypointense on T1 and hypo- or isointense on T2, sometimes with contrast enhancement of the septa, while the cyst content itself remains unchanged [6]. MRI also helps differentiate vertebral hydatidosis from other pathologies such as infectious spondylodiscitis or tumors [7], and assess the intraspinal extension, lesion height, and relationships with neural structures and soft tissues. MRI may also evaluate cyst viability: a high T2 signal suggests ongoing parasitic activity, while a low signal may indicate cyst degeneration or death [6]. In our patient, MRI revealed multivesicular lesions that were hypointense on T1 and hyperintense on T2, with peripheral contrast enhancement-features consistent with viable hydatid cysts. Biologically, eosinophilia may be observed, especially in the case of ruptured or altered cysts, but it is present in only about 40% of cases [8], and was absent in our patient. Immunological tests are often positive during the invasive or complicated phases and are mainly useful for postoperative monitoring. Diagnosis of vertebral hydatidosis is based on a combination of epidemiological, clinical, radiological, and biological findings. Definitive diagnosis is established through histopathological examination of the surgical specimen. Percutaneous biopsy is contraindicated due to the risk of parasitic dissemination [2].

Treatment is primarily surgical. The main objective is to complete removal of hydatid lesions, including both osseous and extraosseous components, while avoiding cyst rupture to minimize dissemination risk [2]. This approach is challenged by the infiltrative, microvesicular nature of the disease within bone tissue, which gives hydatidosis a behavior akin to local malignancy. Surgical resection should ideally include healthy tissue margins, following oncologic resection principles. Depending on the extent of bone loss, reconstruction is often necessary, using bone grafts and sometimes osteosynthesis. Surgery is typically combined with antiparasitic medical therapy. Among benzimidazoles, albendazole has shown the most effectiveness, particularly in visceral sites, and to a lesser extent in osseous localizations [9]. Albendazole is administered at a dose of 10-15 mg/kg/day, in two postprandial doses, in four-week cycles, with a preoperative course followed by several postoperative cycles. In extensive forms, some protocols recommend continuous treatment, though this may be limited by hepatic or hematologic toxicity. In inoperable cases, high-dose medical therapy (up to 800 mg/day) can be continued for 6 to 9 months, aiming to limit lesion progression and reduce dissemination risk [9]. Despite combined management, the prognosis remains guarded, with recurrence rates reaching 30 to 40%, and a reported mortality of 45% to 50% within five years of symptom onset [10]. This underlines the importance of long-term clinical and radiological follow-up. Prevention remains crucial, especially in endemic regions, by breaking the parasitic dog-sheep-human transmission cycle [1]. In our case, urgent surgical decompression combined with postoperative albendazole therapy resulted in complete neurological recovery, with no early recurrence.

## Conclusion

Costo-vertebral hydatidosis is an exceptional and serious entity, often diagnosed at a late stage due to the subtlety of its initial symptoms and its insidious progression. Diagnosis relies on a combination of clinical, epidemiological, radiological, biological, and histological findings. Management remains primarily surgical, with oncologic intent, and is complemented by systemic antiparasitic therapy. Prognosis is mainly influenced by the risk of recurrence and neurological sequelae, warranting a multidisciplinary approach and long-term follow-up. Prevention remains a cornerstone, particularly in endemic areas, to interrupt the transmission cycle and reduce the incidence of severe forms.
